# Effects of Borneol on Pharmacokinetics and Tissue Distribution of Notoginsenoside R1 and Ginsenosides Rg1 and Re in *Panax notoginseng* in Rabbits

**DOI:** 10.1155/2013/706723

**Published:** 2013-03-31

**Authors:** Shixiang Wang, Weijin Zang, Xinfeng Zhao, Weiyi Feng, Ming Zhao, Xi He, Qinshe Liu, Xiaohui Zheng

**Affiliations:** ^1^Department of Pharmacology, School of Medicine, Xi'an Jiaotong University, Xi'an 710061, China; ^2^Key Laboratory of Resource Biology and Biotechnology in Western China, Ministry of Education/College of Life Science, Northwest University, Xi'an 710069, China; ^3^First Affiliated Hospital, School of Medicine, Xi'an Jiaotong University, Xi'an 710061, China; ^4^Shaanxi Provincial People's Hospital, Xi'an 710068, China

## Abstract

The purpose of this study is to investigate the effects of Borneol on the pharmacokinetics of notoginsenoside R1 (NGR1) and the ginsenosides Rg1 (GRg1) and Re (GRe) in *Panax notoginseng*. Reversed phase high-performance liquid chromatography coupled with electrospray ion trap mass spectrometry was employed to determine the concentrations of the three compounds in rabbit plasma. In comparison with rabbits administrated *Panax notoginseng* extract alone, animals simultaneously taking *Panax notoginseng* extract and Borneol exhibited significant differences in pharmacokinetic parameters of NGR1, GRg1, and GRe, such as increasing their bioavailability. Quantities of NGR1, GRg1, and GRe in rabbit tissues were also increased after combining administration of Borneol. In addition, the apparent permeability coefficients (*P*_app_) of NGR1, GRg1, and GRe were raised by Borneol significantly in Caco-2 cells. However, no significant changes were observed in the efflux ratio (Er) of NGR1, GRg1 and GRe. These data indicate that Borneol has the properties of enhancing the intestinal absorption, increasing the distribution, and inhibiting the metabolism of NGR1, GRg1, and GRe. The underlying mechanism might be attributed to the loosening of the intercellular tight junction.

## 1. Introduction

*Panax notoginseng*, also known as sanchi ginseng, is famous in China and other countries for its obvious therapeutic effects on the cardiovascular system [1, 2]. Previous studies have shown that *Panax notoginseng* mainly contained dammarane-type saponins (ginsenosides) including sanchinoside or notoginsenoside which is unique to *Panax notoginseng *[3–6]. Recent researches have revealed various pharmacological effects of notoginsenosides such as blocking Ca^2+^ influx through the receptor, enhancing astrocyte differentiation, and inhibiting vessel restenosis and antifibrotic effects [7–10].

Various methods for the quality control of *Panax notoginseng* and its complex prescription have been reported previously in the literature [11–15]. Among these analytical assays, high-performance liquid chromatography coupled with an ultraviolet visible (UV-Vis) detector or a diode array detector was a common choice for the detection of saponins in *Panax notoginseng*. Setting the detecting wavelength at 190~205 nm due to low absorbance of these compounds in the regular UV region, however, greatly increased the baseline noise and decreased the sensitivity of detection. To address this issue, an evaporative light-scattering detector has been employed for the detection of saponins, resulting in a stable baseline even with a gradient elution [16, 17]. In addition, recent researches have shown that high-performance liquid chromatography coupled with mass spectrometry is a favorable and useful alternative for the detection of saponins in *Panax notoginseng *[18–20].

Borneol, a monoterpenoid component of the medicinal plant such as *Blumea martiniana* and* Clausena dentata *[21–23], is usually used as “Guide drug” in the prescription to guide the bioactive components of herbs to the proper organs to exert a harmonizing effect. A better therapeutic effect has been observed for the combined administration of other herbs, *Panax notoginseng* and *Radix Salvia miltiorrhiza*, and Borneol than the single use of other herbs for the patients with cardiovascular diseases in practice [24, 25]. However, the mechanism underlying the synergistic effect of *Panax notoginseng* and Borneol is still an enigma. In most of the previous studies, pharmacokinetics of saponins in *Panax notoginseng* and its prescriptions were investigated [25–29]. However, little attention has been paid to pharmacokinetics of notoginsenoside R1 (NGR1), ginsenosides Rg1 (GRg1), and Re (GRe), the main active components of *Panax notoginseng*, especially the interactive effects of *Panax notoginseng* and Borneol.

The current study is to investigate the effect of Borneol on the pharmacokinetics of NGR1, GRg1, and GRe in *Panax notoginseng* in rabbits. A sensitive and accurate SPE-HPLC-MS method was established and applied to the pharmacokinetic study of NGR1, GRg1, and GRe via determining their concentrations in rabbit plasma after oral administration of *Panax notoginseng* or *Panax notoginseng* combined with Borneol. In addition, the mechanism underlying the effect of borneol on NGR1, GRg1, and GRe was investigated by vinblastine-selected Caco-2 cells *in vitro*.

## 2. Materials and Methods

### 2.1. Materials and Reagents

NGR1, GRg1, and GRe (purity > 95%) were purchased from the National Institute for the Control of Pharmaceutical and Biological Products of China (Lots nos. 110754-200322; 110703-200322; and 110745-200414, resp.). Borneol (purity > 98%) was supplied by Tianjin Tasly Pharm. Co., Ltd. Caco-2 cells were acquired from Institute of Biochemistry and Cell Biology, Shanghai institute for Biological Sciences, CAS. Transwell plates (pore size 0.4 *μ*m, 24 mm diameter) were purchased from Corning Costar Co. Foetal bovine serum and nonessential amino acids were bought from Gibco-BRL Life Technologies (Paisley, Scotland). Penicillin, streptomycin, trypsin, dimethylsulfoxide (DMSO), and ammonium formate were bought from Sigma Chemical Co. HPLC grade solvents and reagents were obtained from Fisher Scientific Company (Pittsburgh, PA, USA). Ultrapure water (18.2 MΩ) was obtained through a Milli-Q water purification system.

### 2.2. Preparation of Herb Extract

250 grams of *Panax notoginseng* were immersed in an 8-fold ethanol/water (V : V, 70 : 30) solution for 30 min and refluxed twice (1.5 h each time). The suspension was then filtered followed by concentrating to 50 mL to obtain the *Panax notoginseng* extract. The *Panax notoginseng* extract combined with Borneol was prepared by adding 1.42 g Borneol to 50 mL *Panax notoginseng* extract. The concentrations of NGR1, GRg1, and GRe in the extract were determined to be 87.5, 124.6, and 40.2 mg·mL^−1^, respectively, by the HPLC method.

### 2.3. Animals

The ethical use of animals in this study was approved by the Advisory Board on Animal Experiments of the Xi'an Jiaotong University in China. New Zealand rabbits (weight 1.7–2.3 kg) were provided by the Animal Center of Xi'an Jiaotong University. The rabbits were maintained in air-conditioned animal quarters at a temperature of 22 ± 2°C and a relative humidity of 50 ± 10%. The cannula (Terumo, 22 G × 1, i.d. 0.60 × 20 mm) was placed in the central ear artery and used for blood collection. The animals were acclimatized to the facilities for 5 days, and then fasted and had free access to water for 12 h prior to experiment. 

### 2.4. Liquid Chromatographic and Mass Spectrometric Conditions

Liquid chromatography was carried out on an Agilent 1100 HPLC system with an auto sampler, a quaternary pump and a vacuum degasser (Waldoboro, Frankfurt, Germany). Operations were controlled by Agilent Chemstation 4.2 software (Littleforts, Philadelphia, USA). Separations were achieved on a reversed-phase HPLC column (Zorbax SB-C_18_ 150 × 2.1 mm, 5.0 *μ*m particle size). A solution of acetonitrile and water (V : V, 20 : 80) with 0.1% (V : V) ammonium formate was used as the mobile phase. The flow rate was set at 0.3 mL·min⁡^−1^ and the column temperature was 25°C. Under these conditions, NGR1, GRg1, and GRe in plasma samples were separated efficiently without any interferences.

MS^n^ detection was performed on an Agilent SL trap MS system (Waldoboro, Frankfurt, Germany). The ion source-dependent (electrospray ionization) conditions were the same for all analyses with a spraying voltage of −4500 V in the negative ion mode. The pressure of the nebulizing gas (nitrogen) was set at 35 p.s.i. The flow rate of the drying gas (nitrogen) was set at 7.0 L·min^−1^ with the temperature of 325°C. The collision gas (He) for the MS^n^ mode at trap was set at flow of 4 (instrument unit). The voltage of the capillary was set at 4000 V, and its end plate offset was −500 V. Scan range was from 500 to 1500 *m/z*. 

### 2.5. Preparation of Calibration Standard Working Solutions

Primary stock solutions of 0.28 mg·min^−1^ NGR1, 0.30 mg·min^−1^ GRg1 and 0.72 mg·min^−1^ GRe were prepared in methanol. Working standard solutions of NGR1, GRg1, and GRe were prepared by diluting the aliquots of the primary solution with methanol. The solutions were stored at 4°C in glass tubes until further use. 

### 2.6. Extraction of Sample

Frozen plasma and tissue samples were thawed in a water bath at 37°C and were then vortexed followed by centrifuging at 5000 r·min^−1^ for 5 min. An aliquot of 1.0 mL of the supernatant from each sample was loaded onto C_18_ Bond Elute Solid phase extraction (SPE) cartridges (1000 mg, 1 cc reservoir, Varian, Harbor City, CA, USA) pretreated with 2.0 mL hexane, isopropanol, methanol, and water, sequentially. The SPE cartridges were then washed with 1.0 mL water, 20% methanol/water solution, 40% methanol/water solution, and 60% methanol/water solution, sequentially. Finally, analytes were eluted twice with 1.0 mL of 70% methanol/water solution. The eluant was evaporated to dryness under nitrogen. The residues were then reconstituted in 1.0 mL mobile phase. An aliquot of 10 *μ*L was injected into the LC-MS system.

### 2.7. Calibration Procedure

Samples calibration standards containing 0.28, 0.56, 2.8, 5.6, 14.0, 28.0, and 56.0 *μ*g·min^−1^ of NGR1, 0.30, 0.60, 3.0, 6.0, 15.0, 30.0, and 60.0 *μ*g·min^−1^ of GRg1, and 0.36, 0.72, 3.6, 7.2, 18.0, 36.0, and 72.0 *μ*g·min^−1^ of GRe were freshly prepared daily by diluting the working standard solution with blank sample. The calibration curve was then obtained by plotting the peak areas of the extracted ion current versus the concentrations of the standards using weighted linear regression. The results showed that the linear range of NGR1, GRg1, and GRe was 0.28–56.0, 0.30–60.0, and 0.36–72.0 *μ*g·min^−1^, respectively. 

### 2.8. Method Validation

Validation of the proposed method included assessment of the calibration curve performance, as well as accuracy and precision of the method, and stability of the analytes at various test conditions.

The precision of the assay was determined for the quality control (QC) plasma and tissue samples by replicate analyses of three levels of concentration at 0.5, 5.0, and 35.0 *μ*g·min^−1^ for NGR1, 0.4, 3.0, and 40.0 *μ*g·min^−1^ for GRg1, and 0.8, 8.0, and 48.0 *μ*g·min^−1^ for GRe. Intraday precision and accuracy were determined via repeated analysis of the QC plasma and tissue samples within one day (*n* = 5). Interday precision and accuracy were determined via repeated analysis on five consecutive days. The concentration of each sample was determined using the prepared calibration curve and analyzed on the same day. All stabilities were evaluated at different concentration levels. Short-term stability of NGR1, GRg1, and GRe were assessed by analyzing QC samples kept at 4°C for 4–24 h. Freeze-thaw stability was evaluated at three consecutive freeze-thaw cycles. Long-term stability was studied by analyzing samples during a period of 8 weeks of storage at −70°C.

### 2.9. Pharmacokinetics Study

Eighteen rabbits were randomly divided into three groups of 6 subjects and were orally given 3.0 mL·kg^−1^ normal saline, 3.0 mL·kg^−1^ *Panax notoginseng* extract, and 3.0 mL·kg^−1^ *Panax notoginseng* extract combined with Borneol, respectively. Plasma samples were collected in heparinized tubes from the central ear artery at 0.0, 5.0, 10.0, 20.0, 30.0, 45.0, 60.0, 75.0, 90.0, 120.0, 180.0, 300.0 and 480.0 min after dose. After each sampling, the same volume of 0.9% saline solution was injected from the ear vein to compensate the loss of blood. The plasma obtained was frozen at −70°C for storage and was processed prior to analysis with the proposed method as described in Section 2.6.

### 2.10. Tissue Distribution Study

One group of rabbits (*n* = 18) was orally administered a dose of 3.0 mL·kg^−1^ *Panax notoginseng* extract, while another group of rabbits (*n* = 18) was orally given 3.0 mL·kg^−1^ *Panax notoginseng* extract combined with Borneol. At 0.5, 1, and 3 h after administration, blood samples were collected from the central ear artery of six rabbits from each group, and the heart, liver, lung, kidney, and brain were immediately removed after animals were sacrificed by decapitation. An accurately weighed amount of tissue (1 g) was collected to be rinsed, dried, minced, and homogenized (400 r·min^−1^) in normal saline (1.5 mL). All of the samples were stored at −70°C and were processed prior to analysis with the proposed method as described in Section 2.6.

### 2.11. Transport Studies

The Caco-2 cells were cultured in Dulbecco's modified Eagle's medium (DMEM) supplemented with 20% foetal bovine serum, 1% nonessential amino acids and penicillin-streptomycin, at 37°C in an atmosphere with a relative humidity of 95% and a CO_2_ flow of 5%. Medium was replaced every 2-3 days. When the cell monolayer reached 80% confluence, the cells were detached with a solution of 0.02% EDTA and 0.25% trypsin. The vinblastine-selected Caco-2 cells were cultivated in the presence of 10 nM vinblastine to induce P-glycoprotein (P-gp) expression. The culture medium was changed to a fresh medium without vinblastine 24 h before experiments, and the cells were used between passages 25 and 46. Prior to the transport study, cytotoxicity of NGR1, GRg1, GRe, and Borneol toward Caco-2 cells was determined using MTT assays. Noncytotoxic concentrations of 500 *μ*M NGR1, GRg1, GRe, and 200 *μ*M Borneol (dissolved in DMSO) were chosen for transport study.

In transport studies, vinblastine-selected Caco-2 cells were seeded on polycarbonate filter of transwells for 18–21 days before starting transport study, and the monolayers with the transepithelial electrical resistance (TEER) values greater than 300 *Ω*cm^2^ were used. Caco-2 monolayers were rinsed twice with Hanks' balanced salt solution (HBSS) and preincubated in HBSS at 37°C for 30 min before starting experiments. To start the experiments, 500 *μ*M of NGR1, GRg1, and GRe in final concentrations were added to the donor side with or without 200 *μ*M Borneol and then incubated at 37°C. An aliquot of 0.1 mL samples were withdrawn from receiver chambers at 0, 30, 60, 90, and 120 min after the loading. After each sampling, 0.1 mL of HBSS was added to the receiver chamber to maintain a constant volume. All the experiments were performed five times in duplicate. The collected samples were stored at −20°C until HPLC analysis. During the above transport studies, the TEER values were also monitored before and at the end of each experiment. Apparent permeability coefficients (*P*_app_) were then calculated according to the following equation:

(1) $$\begin{array}{c} P_{\text{app}}=\frac{\left(dC/dt\times V\right)}{\left(A\times C_{0}\right)}, \end{array}$$

where *dC*/*dt* is the rate of the test compound appearing in the receiver chamber, *V* is the volume of the solution in the receiver chamber, *A* is the cell monolayer surface area, and *C*_0_ is the initial concentration of the test compound added in the donor chamber.

The efflux ratio (Er) was calculated using the following equation:

(2) $$\begin{array}{c} \text{Er}=\frac{P_{\text{app}}\left(\text{basolateral-apical}\right)}{P_{\text{app}}\left(\text{apical-basolateral}\right)}. \end{array}$$

### 2.12. Statistical Analysis

Statistical analysis of the biological data was performed using the Student's *t*-test. The drug analysis system 2.0 (DAS 2.0, T.C.M., Shanghai, China) was used to calculate the pharmacokinetic parameters, such as the area under curve (AUC), the maximum plasma concentration (*C*_max⁡_), the time needed to reach the maximum plasma concentration (*t*_max⁡_) and the half-life of absorption, and distribution and elimination (*t*_1/2*K*_*a*__,   *t*_1/2*α*_,   *t*_1/2*β*_). 

## 3. Results and Discussion

### 3.1. Method Validation

#### 3.1.1. Specificity

**Figure 1:**
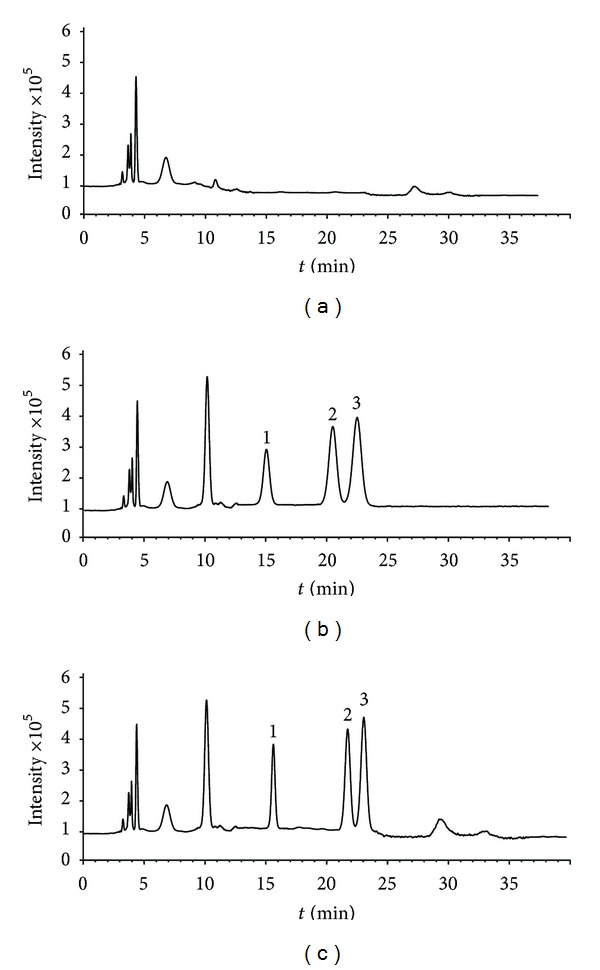
HPLC-MS ion chromatograms of plasma samples. (a) blank plasma samples; (b) plasma standard solutions of 5.0 *μ*g·mL^−1^ NGR1, 3.0 *μ*g·mL^−1^ GRg1, and 8.0 *μ*g·mL^−1^ GRe; (c) plasma samples after administration of *Panax notoginseng* extract at a dose volume of 3.0 mL·kg^−1^.

The base peaks of each mass spectrum for NGR1, GRg1, and GRe were observed during the infusion of the standard solution in negative mode. Three [M-H]^−^  precursor ions, *m/z* 931.6 [M-H]^−^  for NGR1, *m/z* 799.5 [M-H]^−^  for GRg1, and *m/z* 945.1 [M-H]^−^, were subjected to collision-induced dissociation (CID). The product ions were recorded as *m/z* 799.4 [M-H-Xyl]^−^, 637.3 [M-H-Glc]^−^, and *m/z* 799.2 [M-H-Rha]^−^, respectively. Mass transition patterns, *m/z* 931.6 → 799.4, *m/z* 799.5 → 637.3, and *m/z* 945.1 → 799.2, were selected to monitor NGR1, GRg1, and GRe. Representative HPLC-MS ion chromatograms of blank plasma samples, plasma standard solutions of 5.0 *μ*g·mL^−1^ NGR1, 3.0 *μ*g·mL^−1^ GRg1 and 8.0 *μ*g·mL^−1^ GRe as well as plasma samples after administration of *Panax notoginseng* extract at a dose volume of 3.0 mL·kg^−1^ are shown in Figure 1. No endogenous peaks were found to be coeluted with the analytes, indicating high specificity of the proposed method. 

#### 3.1.2. Calibration Curve Performance

The calibration curves were created by plotting the peak areas of NGR1, GRg1, and GRe to their various concentrations in the spiked plasma and tissue standards. A weighted (1/[nominal concentration]) least-squares linear regression of the type *y* = *bx* + *a* was used to fit the curves (Table 1). The lowest correlation coefficient of determination (*r* ^2^) among the five calibration curves of NGR1, GRg1, and GRe were between 0.9982 and 0.9996. Thus, the calibration curves exhibited good linearity within the chosen range. 

#### 3.1.3. Limit of Detection and Quantitation

The limit of detection (LOD) was estimated as the amount of NGR1, GRg1, and GRe, which caused a signal three times that of noise (*S*/*N* = 3/1). The LOD was determined to be 0.57, 0.30, and 0.24 ng·mL^−1^ in lung and liver, and 0.28, 0.15, and 0.12 ng·mL^−1^ in plasma and other tissues, respectively. The lower limit of quantitation (LLOQ) was defined as the lowest concentration with the accuracy and precision better than 20% and a signal to noise ratio of >10. The LLOQ for NGR1, GRg1, and GRe were determined to be 1.8, 1.0, and 0.8 ng·mL^−1^ in lung and liver and 1.0, 0.5, and 0.4 ng·mL^−1^ in plasma and other tissues, respectively.

#### 3.1.4. Accuracy and Precision

Data for intraday and interday precision and accuracy assessed by analyzing QC samples at different concentrations are presented in Table 2. The results suggested that the method was adequately accurate and reproducible for the determination of NGR1, GRg1, and GRe in rabbit plasma and tissues.

#### 3.1.5. Extraction Recovery and Stability

The extraction recovery analysis was conducted with NGR1, GRg1, and GRe spiked biosamples at three QC levels and calculated by comparing the NGR1, GRg1, and GRe peak areas in extracted biosamples with those found by direct injection of standard solutions at the same concentration. The mean recoveries of NGR1, GRg1, and GRe in plasma and tissue samples at three different concentrations were above 90.0% (Table 2).

The stability studies were performed by evaluating small variations in three different conditions. The results were expressed as the percentage of initial content of NGR1, GRg1, and GRe in the freshly treated samples, suggesting that NGR1, GRg1, and GRe showed no significant change in plasma and tissue samples (Table 3).

### 3.2. Pharmacokinetics Study

**Figure 2:**
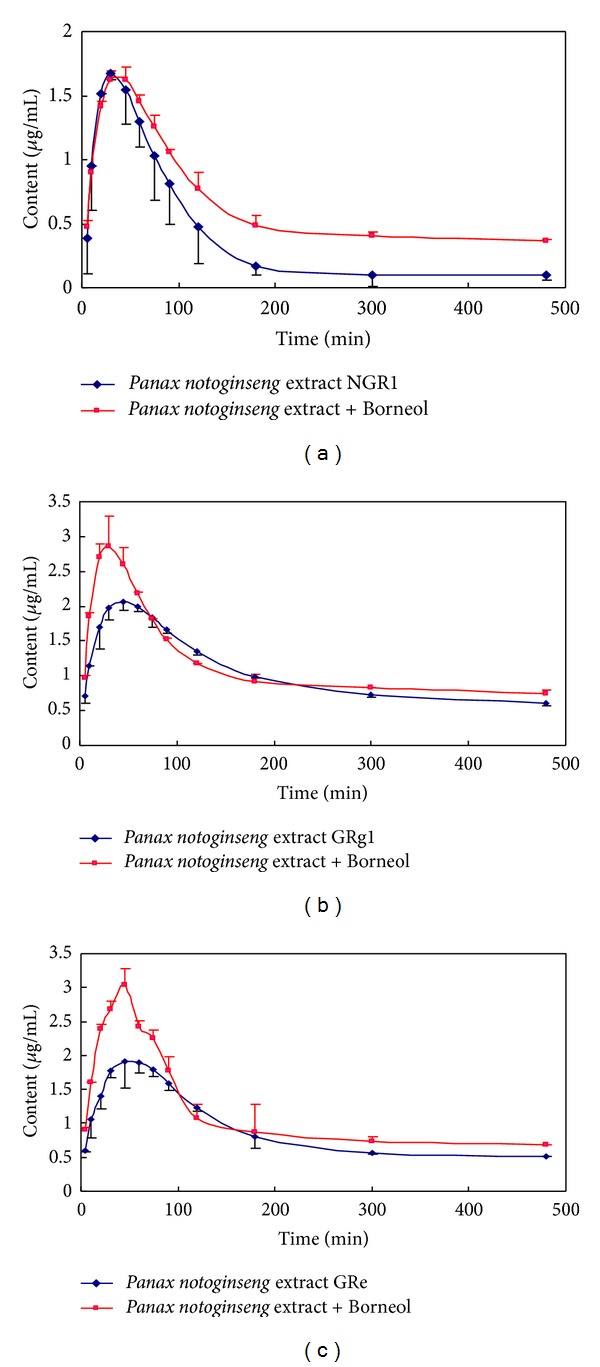
Plasma concentration-time curves of NGR1 (a), GRg1 (b), and GRe (c) after administration of *Panax notoginseng and Panax notoginseng* combined with Borneol extracts in rabbit, respectively. The dose volume was 3.0 mL·kg^−1^  and the fitted curves were obtained by analyzing the plasma concentration-time data with the Program DAS 2.0. *◆* rabbits administered *Panax notoginseng *extract; ■ rabbits administered *Panax notoginseng *combined with Borneol.

After oral administration of *Panax notoginseng* or *Panax notoginseng* combined with Borneol, the plasma concentrations of NGR1, GRg1, and GRe were determined by the described LC/MS/MS method.  Figure 2 showed the plasma concentration-time curves of NGR1, GRg1, and GRe following ingestion of *Panax notoginseng* or *Panax notoginseng* combined with Borneol (*n* = 6). The statistical results through DAS 2.0 indicated that the plasma drug concentration-time course of the three compounds in rabbits confirmed the 2-compartment open models. The corresponding regression pharmacokinetic parameters were shown in Table 4.

It can be noted that the highest values of GRg1 were approximately the same as the values of GRe. This partly ascribed to the similar chemical properties of the two compounds. In addition, the increasing tendency of total distribution volume (*V/F*) for NGR1 was similar to that for GRg1 and GRe. However, the highest values of NGR1 parameters were different from the values of GRg1 and GRe. 

Combined with Borneol, the values of *t*_1/2*α*_ decreased but the AUC values increased obviously, which indicated that Borneol improved the absorption rate and bioavailability of NGR1, GRg1, and GRe. In addition, the decreased value of *K*_10_ and the increased value of *K*_12_ indicated that Borneol slowed down the clearance speed of NGR1, GRg1, and GRe, but increased the transferring speed of these compounds from the central compartment to the peripheral compartment. The increase in *V/F* indicated that NGR1, GRg1, and GRe transferred from the blood to the tissues, but the transfer speed was different.

In contrast to the pharmacokinetics of NGR1 in the *Panax notoginseng* group and the *Panax notoginseng* combined with Borneol group, the value of *K*_*a*_ was reduced, *t*_1/2*K*_*a*__ was increased, **β** was reduced, and *t*_1/2*β*_ was increased, indicating that the absorption and the clearance speed of NGR1 in the *Panax notoginseng* combined with Borneol group were reduced. Compared with the pharmacokinetic parameters of GRg1 and GRe in these two groups, the absorption rate was increased and the absorption time was reduced, while the clearance speed was constant in the *Panax notoginseng* group and the *Panax notoginseng* combined with Borneol group. In these comparisons, Borneol had different effects on the values of *K*_*a*_, *t*_1/2*K*_*a*__, **β**, *t*_1/2*β*_, and *K*_12_ of NGR1, GRg1, and GRe.

### 3.3. Tissue Distribution Study

As listed in Table 5, compared with other organs, NGR1 and GRe levels in heart as well as GRg1 level in lung were high, but NGR1 and GRg1 levels in brain as well as GRe level in lung were low at 0.5, 1.0, and 3.0 h in *Panax notoginseng *group. The highest levels of NGR1, GRg1, and GRe were observed at 1.0 h in heart, liver, lung, and brain, meanwhile the drug concentration in kidney decreased at 1.0 h. For Borneol combined with *Panax notoginseng*, the three saponins levels were all increased markedly in the tissues with peak levels observed at 1.0 h in the tissues except kidney. The levels of NGR1 in heart, liver, brain, lung and kidney were 3.90-, 6.36-, 3.82-, 6.82-, and 2.3-fold higher than the plasma concentrations, respectively. The GRg1 levels in these tissues were 12.40-, 27.09-, 11.77-, 8.17-, and 7.77-fold higher than the plasma concentrations, respectively. The GRe levels in these tissues were 1.35-, 1.97-, 1.14-, 1.24-, and 1.0-fold higher than the plasma concentrations, respectively. These data indicate that Borneol could increase the levels of NGR1, GRg1, and GRe in the tissues. 

### 3.4. Transport Studies

According to the classification method proposed by Yee [30], the permeabilities less than 10^−6^ cm/s correspond to substances with low absorption (<30%), permeabilities between 10^−6^ cm/s and 10^−5^ cm/s correspond to substances with moderate absorption (30–70%), and permeabilities more than 10^−5^ cm/s correspond to substances with high absorption (>70%). As showed in Table 6, the *P*_app_ values of NGR1, GRg1, and GRe were less than 10^−6^ cm/s, indicating that NGR1, GRg1, and GRe presented the poor membrane permeabilities and low bioavailabilities in Caco-2 monolayers. The efflux ratios (Er) of NGR1, GRg1, and GRe were within the range of 1.0-1.1, suggesting that there was no significant difference between the permeability in apical-to-basolateral and that in basolateral-to-apical directions, and implying that NGR1, GRg1, and GRe seemed not to be substrates of P-gp. However, it was reported that metabolic inhibitor KCN and P-gp inhibitor verapamil could increase GRg1 concentration within the cells, and the efflux of Rg1 was energy-dependent and P-gp was likely to be involved [31]. Its precise mechanism still needs to be investigated in further work.

**Figure 3:**
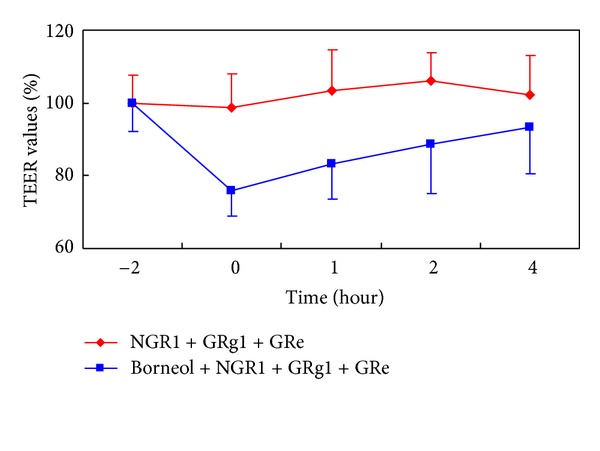
Effect of Borneol on TEER values of the Caco-2 cell monolayers. The Caco-2 cell monolayers were pretreated 2 h with 500 *μ*M of NGR1, GRg1, and GRe, or the three saponins plus 200 *μ*M Borneol. At time point 0, the monolayers were washed with buffered DMEM (pH 7.4), and then incubated at 37°C for 4 h.

Borneol is used as a “Guide drug” in traditional Chinese medicine, enhancing the expected functions of bioactive components from other herbs in the complex prescription through increasing bioavailability. Other research groups have found that Borneol could obviously loosen the intercellular tight junction, increase the number and volume of pinocytosis vesicles [32, 33], promote the fluidity of membrane and the permeability of bilayer lipid membrane *in vitro *[34], and inhibit the function of P-gp on cell membrane [35]. In this experiment, we found that Borneol increased the *P*_app_ (apical-to-basolateral) and *P*_app_ (basolateral-to-apical) values of NGR1, GRg1 and GRe significantly, by 2.9-, 2.6-, and 2.3-fold and 2.9-, 2.6-, and 2.4-fold, respectively. Meanwhile, TEER values of the monolayers decreased reversibly to about 23% (Figure 3). These data imply that Borneol may open the paracellular spaces between cells and enhance permeability of NGR1, GRg1, and GRe. However, no significant changes in Er of NGR1, GRg1, and GRe were observed, indicating that the three saponins are not substrates of P-gp. We may therefore suppose that Borneol could loosen the intercellular tight junction and enhance permeability of NGR1, GRg1, and GRe, which is probably the main reason why Borneol enhances the bioavailability of NGR1, GRg1, and GRe.

## 4. Conclusion

In summary, the present study showed that after combined oral administration to rabbits with *Panax notoginseng,* Borneol significantly changed the pharmacokinetic parameters of NGR1, GRg1, and GRe, the main active compounds in *Panax notoginseng*. The possible mechanism was that Borneol could loosen the intercellular tight junction and enhance permeability of NGR1, GRg1, and GRe. Our results might help in guiding the clinic use of Borneol and other herbs in traditional Chinese medicine.

## Figures and Tables

**Table 1:** Calibration curves for the analysis of NGR1, GRg1, and GRe in rabbit plasma and tissue.

Biosample	Calibration curves	Correlation coefficient (*r* ^2^)	Linear range (*μ*g/mL)
NGR1			
Plasma	*Y* = 356948*X* + 1.0076	0.9990	0.280–56.0
Heart	*Y* = 397087*X* − 9.5861	0.9992	
Liver	*Y* = 389965*X* − 9.4869	0.9990	
Brain	*Y* = 390069*X* − 8.4391	0.9996	
Lung	*Y* = 379924*X* − 8.5585	0.9992	
Kidney	*Y* = 386942*X* − 9.2368	0.9996	
GRg1			
Plasma	*Y* = 358992*X* − 3.0221	0.9988	0.307–60.4
Heart	*Y* = 356409*X* − 2.6782	0.9982	
Liver	*Y* = 367748*X* − 3.4734	0.9986	
Brain	*Y* = 362745*X* − 2.9939	0.9996	
Lung	*Y* = 359638*X* − 4.1365	0.9990	
Kidney	*Y* = 364720*X* − 4.5526	0.9990	
GRe			
Plasma	*Y* = 293769*X* − 1.605	0.9996	0.362–54.3
Heart	*Y* = 284093*X* + 3.8607	0.9988	
Liver	*Y* = 279365*X* + 3.9834	0.9992	
Brain	*Y* = 287562*X* + 4.1262	0.9986	
Lung	*Y* = 285328*X* + 3.9967	0.9988	
Kidney	*Y* = 294563*X* + 4.0062	0.9990	

**Table 2:** The interday and intraday precision and accuracy of the method for the determination of NGR1, GRg1, and GRe (*n* = 5).

Biosample	QC conc (*μ*g · mL^−1^)	Intraday		Interday		Extraction recovery	
		Precision (R.S.D %)	Accuracy (mean %)	Precision (R.S.D %)	Accuracy (mean %)	Mean ± S.D.	R.S.D %
NGR1							
	0.5	10.4	96.0	13.0	92.0	93.5 ± 4.7	5.1
Plasma	5.0	6.7	102.0	11.1	94.0	91.7 ± 3.5	3.8
	35.0	4.2	97.4	5.4	103.7	95.7 ± 7.5	7.9
	0.5	6.3	92.5	8.4	104.9	97.8 ± 6.8	7.0
Heart	5.0	8.3	91.8	9.3	98.9	107.4 ± 14.6	8.4
	50.0	4.9	100.3	5.3	106.1	96.9 ± 9.8	10.6
	0.5	8.7	98.1	10.6	95.8	92.7 ± 7.8	8.4
Liver	5.0	7.5	91.4	8.3	96.6	102.1 ± 5.0	4.9
	50.0	7.1	99.6	7.8	105.6	95.5 ± 7.1	7.4
	0.5	9.5	90.0	6.3	108.3	92.3 ± 9.3	10.4
Brain	5.0	4.2	108.3	9.7	95.7	99.2 ± 7.7	7.8
	50.0	3.7	103.5	7.9	95.6	92.7 ± 4.8	5.3
	0.5	7.4	94.2	13.1	103.7	105.3 ± 8.9	8.5
Lung	5.0	12.1	98.6	4.8	105.5	95.0 ± 8.2	8.6
	50.0	6.7	105.8	10.2	95.4	90.9 ± 9.2	10.1
	0.5	8.2	90.2	5.4	90.8	101.7 ± 8.5	6.2
Kidney	5.0	11.1	91.4	3.8	98.4	92.7 ± 3.7	4.0
	50.0	5.9	90.5	7.8	91.3	90.5 ± 5.1	5.6
GRg1							
	0.4	14.3	105.1	13.5	92.5	103.2 ± 4.6	4.5
Plasma	3.0	4.4	90.0	9.0	103.3	92.4 ± 7.5	8.1
	40.0	4.6	95.3	4.1	98.3	93.2 ± 5.0	5.4
	0.4	10.2	97.4	6.4	96.3	95.7 ± 9.8	10.2
Heart	3.0	4.4	93.9	7.5	94.7	99.2 ± 7.7	7.8
	40.0	6.2	101.3	10.4	104.6	106.3 ± 8.7	8.2
	0.4	9.9	97.2	12.3	98.4	90.5 ± 7.1	7.9
Liver	3.0	12.4	92.5	7.8	96.2	95.7 ± 10.0	10.5
	40.0	6.3	90.9	6.8	102.5	91.8 ± 11.3	12.3
	0.4	8.9	108.9	13.2	90.4	104.8 ± 6.8	6.5
Brain	3.0	6.1	96.3	8.4	94.3	98.2 ± 5.6	5.7
	40.0	7.3	101.8	9.3	103.1	97.9 ± 8.1	8.3
	0.4	11.8	91.9	8.8	92.8	93.4 ± 8.0	8.6
Lung	3.0	8.4	98.0	7.5	91.9	96.1 ± 4.7	4.9
	40.0	6.2	104.7	5.4	108.2	96.8 ± 7.0	7.3
	0.4	5.4	92.8	10.2	98.7	93.3 ± 9.8	10.5
Kidney	3.0	7.2	91.5	5.4	90.4	95.1 ± 3.6	3.8
	40.0	6.1	99.2	6.7	92.5	90.5 ± 6.9	7.6
GRe							
	0.8	8.4	103.7	11.5	97.5	91.2 ± 6.1	6.7
Plasma	8.0	6.6	92.5	6.2	107.5	90.8 ± 7.2	7.9
	48.0	4.2	104.7	3.8	102.9	98.1 ± 7.7	7.8
	0.8	5.4	95.8	5.8	92.6	105.3 ± 9.8	9.3
Heart	8.0	6.8	99.4	8.3	101.5	99.2 ± 5.7	5.8
	48.0	5.5	109.1	6.7	103.8	93.1 ± 8.2	8.8
	0.8	9.1	96.8	10.8	98.3	97.2 ± 10.2	10.5
Liver	8.0	6.8	94.5	9.6	96.1	94.4 ± 6.2	6.6
	48.0	9.1	96.8	8.4	98.0	106.2 ± 7.7	7.3
	0.8	12.1	91.0	7.7	99.5	93.3 ± 6.3	6.7
brain	8.0	8.2	109.5	13.2	104.3	95.8 ± 8.9	9.3
	48.0	7.3	104.8	9.4	93.9	94.5 ± 6.4	6.8
	0.8	7.8	92.6	9.8	96.3	102.9 ± 9.7	9.4
Lung	8.0	8.5	96.4	11.4	101.6	95.7 ± 7.3	7.7
	48.0	4.7	95.9	7.8	94.8	101.7 ± 7.0	6.9
	0.8	6.1	91.8	8.5	92.8	98.1 ± 7.9	8.1
Kidney	8.0	3.3	92.2	9.0	90.6	90.2 ± 4.2	4.7
	48.0	8.9	96.4	4.5	95.4	97.4 ± 5.8	6.0

**Table 3:** Stability of of NGR1, GRg1, and GRe in plasma samples and tissue homogenates of rabbits (*n* = 6).

Biosample	QC conc (*μ*g · mL^−1^)	Remaining (mean ± S.D.)		
		Short-term stability	Long-term stability	Freeze-thaw stability
NGR1				
	0.5	98.0 ± 6.2	100.0 ± 8.7	90.4 ± 5.9
Plasma	5.0	96.0 ± 8.4	94.0 ± 6.8	92.8 ± 6.4
	35.0	99.8 ± 3.3	97.0 ± 5.3	96.6 ± 5.5
	0.5	98.2 ± 6.7	103.6 ± 6.5	92.8 ± 6.8
Heart	5.0	94.8 ± 9.5	92.9 ± 4.2	102.5 ± 10.6
	50.0	100.3 ± 7.5	96.6 ± 6.7	96.7 ± 8.7
	0.5	95.1 ± 3.1	92.6 ± 9.1	98.9 ± 7.9
Liver	5.0	94.3 ± 6.7	96.6 ± 6.4	103.8 ± 8.2
	50.0	95.4 ± 8.6	100.9 ± 5.6	99.0 ± 5.0
	0.5	92.3 ± 9.4	101.8 ± 6.8	92.8 ± 6.9
Brain	5.0	98.4 ± 4.4	90.0 ± 9.5	92.2 ± 8.5
	50.0	103.5 ± 9.2	99.0 ± 5.3	95.2 ± 11.4
	0.5	91.2 ± 5.4	97.6 ± 6.8	95.8 ± 9.8
Lung	5.0	96.8 ± 8.3	89.8 ± 4.6	91.9 ± 9.3
	50.0	95.6 ± 5.9	91.6 ± 9.8	96.9 ± 7.5
	0.5	92.4 ± 4.3	91.1 ± 5.4	92.2 ± 7.5
Kidney	5.0	95.8 ± 5.6	94.3 ± 2.8	95.4 ± 8.9
	50.0	90.8 ± 3.7	96.8 ± 9.5	80.9 ± 6.7
GRg1				
	0.4	102.0 ± 5.7	92.0 ± 5.7	96.0 ± 3.9
Plasma	3.0	94.0 ± 5.9	86.0 ± 9.7	91.1 ± 8.2
	40.0	102.8 ± 9.6	104.6 ± 5.5	103.6 ± 7.0
	0.4	97.8 ± 8.2	98.3 ± 4.4	91.3 ± 6.1
Heart	3.0	96.3 ± 8.4	95.1 ± 5.2	90.8 ± 9.7
	40.0	92.5 ± 4.8	103.2 ± 6.4	102.3 ± 6.6
	0.4	92.5 ± 7.2	95.6 ± 9.7	93.5 ± 8.9
Liver	3.0	94.8 ± 6.4	92.8 ± 6.8	91.9 ± 2.4
	40.0	93.8 ± 8.4	94.7 ± 5.8	93.8 ± 3.8
	0.4	97.5 ± 5.6	90.2 ± 8.3	95.6 ± 5.7
Brain	3.0	93.7 ± 7.2	105.4 ± 8.7	90.9 ± 6.4
	40.0	90.6 ± 6.4	98.7 ± 7.8	98.3 ± 6.1
	0.4	96.1 ± 4.9	90.5 ± 9.8	96.2 ± 4.5
Lung	3.0	99.0 ± 7.9	92.4 ± 3.7	92.3 ± 4.8
	40.0	92.4 ± 9.2	98.9 ± 5.4	98.0 ± 6.7
	0.4	91.5 ± 6.5	90.8 ± 6.1	90.5 ± 7.8
Kidney	3.0	95.9 ± 4.5	91.6 ± 11.2	94.7 ± 3.7
	40.0	102.1 ± 5.7	95.7 ± 4.6	91.2 ± 10.6
GRg1				
	0.4	101.6 ± 9.2	96.0 ± 7.9	105.0 ± 5.8
Plasma	3.0	91.8 ± 7.8	86.0 ± 7.2	103.7 ± 8.5
	40.0	97.3 ± 9.5	104.6 ± 6.3	103.1 ± 7.9
	0.4	98.4 ± 3.9	92.4 ± 5.5	91.9 ± 7.8
Heart	3.0	102.5 ± 6.7	95.3 ± 4.2	95.5 ± 8.3
	40.0	92.8 ± 4.9	93.6 ± 6.8	97.8 ± 5.2
	0.4	95.5 ± 6.1	95.9 ± 8.4	97.2 ± 5.7
Liver	3.0	100.5 ± 3.7	92.4 ± 9.2	93.9 ± 6.5
	40.0	95.8 ± 6.3	96.1 ± 4.5	96.7 ± 7.9
	0.4	92.2 ± 3.8	93.7 ± 6.4	90.5 ± 6.8
Brain	3.0	96.7 ± 5.3	96.0 ± 3.5	92.9 ± 5.4
	40.0	93.8 ± 8.5	97.0 ± 6.8	95.4 ± 6.7
	0.4	96.2 ± 3.9	92.4 ± 6.4	90.9 ± 5.9
Lung	3.0	91.7 ± 5.8	93.5 ± 5.3	97.2 ± 5.8
	40.0	96.4 ± 7.3	97.7 ± 8.7	93.8 ± 7.3
	0.8	93.6 ± 5.7	96.3 ± 4.2	90.7 ± 3.4
Kidney	8.0	95.2 ± 4.2	98.3 ± 7.2	93.8 ± 12.2
	48.0	92.8 ± 3.8	92.9 ± 9.0	92.3 ± 5.8

**Table 4:** The statistical parameters of NGR1, GRg1, and GRe after administration of *Panax notoginseng* and *Panax notoginseng* combined with Borneol.

Parameters	*Panax notoginseng *			*Panax notoginseng* with Borneol		
	NGR1	GRg1	GRe	NGR1	GRg1	GRe
*α* (min^−1^)	0.018 ± 0.008	0.018 ± 0.005	0.020 ± 0.004	0.024 ± 0.005	0.031 ± 0.011	0.027 ± 0.008
*β* (min^−1^)	0.014 ± 0.003	0.010 ± 0.007	0.010 ± 0.002	0.010 ± 0.003*	0.010 ± 0.001	0.010 ± 0.001
*t*_1/2*α*_ (min)	38.5 ± 4.5	38.5 ± 2.5	35.0 ± 1.9	28.4 ± 3.2**	22.3 ± 3.1**	25.2 ± 2.4**
*t*_1/2*β*_ (min)	47.9 ± 8.1	69.3 ± 12.0	69.3 ± 5.8	69.3 ± 5.2**	69.3 ± 10.7	69.3 ± 15.2
V/F (L · kg^−1^)	27.3 ± 8.6	24.5 ± 4.5	20.0 ± 4.7	58.8 ± 6.9**	35.9 ± 8.7*	31.1 ± 6.9*
CL/F (L · min^−1^ · kg^−1^)	0.488 ± 0.091	0.123 ± 0.067	0.150 ± 0.030	0.506 ± 0.027	0.119 ± 0.040	0.143 ± 0.054
AUC_0–*t*_ (mg · L^−1^ · min^−1^)	162.1 ± 42.7	494.8 ± 46.5	424.9 ± 79.6	306.3 ± 82.9**	545.1 ± 51.7	525.1 ± 101.3
AUC_0–*∞*_ (mg · L^−1^ · min^−1^)	164.0 ± 51.8	651.9 ± 73.9	534.7 ± 123.8	395.3 ± 101.4**	1674.6 ± 148.2**	1400.6 ± 251.9**
*K *_ 10_ (min^−1^)	0.018 ± 0.005	0.005 ± 0.001	0.007 ± 0.003	0.009 ± 0.002*	0.003 ± 0.00	0.005 ± 0.001
*K *_ 12_ (min^−1^)	0.000 ± 0.000	0.011 ± 0.003	0.012 ± 0.004	0.015 ± 0.005**	0.025 ± 0.005**	0.022 ± 0.005**
*K *_ 21_ (min^−1^)	0.014 ± 0.007	0.012 ± 0.003	0.011 ± 0.005	0.011 ± 0.002	0.013 ± 0.001	0.011 ± 0.003
*K *_*a*_ (min^−1^)	0.060 ± 0.004	0.039 ± 0.004	0.025 ± 0.004	0.037 ± 0.005**	0.051 ± 0.010*	0.034 ± 0.003**
*C*_max⁡_ (mg · L^−1^)	2.12 ± 0.46	2.36 ± 0.15	1.92 ± 0.22	1.62 ± 0.30	2.87 ± 0.34**	3.04 ± 0.24**
*T*_max⁡_ (min)	45.0 ± 9.8	30.0 ± 5.2	45.0 ± 0.0	30.0 ± 8.0*	30.0 ± 0.0	45.0 ± 13.4
*t*_1/2*Ka*_(min)	11.6 ± 2.4	17.8 ± 2.4	27.7 ± 3.7	18.8 ± 3.1**	13.5 ± 4.6	20.3 ± 4.2**
*T*_lag_ (min)	2.38 ± 0.49	0 ± 0	0 ± 0	1.04 ± 0.21**	1.25 ± 0.34**	0.61 ± 0.47*

*P < 0.05, **P < 0.01 compared with *Panax notoginseng*.

**Table 5:** Drug concentrations in rabbit tissues after administration of *Panax notoginseng* and *Panax notoginseng* combined with Borneol (*n* = 6).

Time (*h*)	Biosample	Concentration (*μ*g · g^−1^)					
		*Panax notoginseng *			*Panax notoginseng* with Borneol		
		NGR1	GRg1	GRe	NGR1	GRg1	GRe
0.5	Heart	3.90 ± 0.53	2.21 ± 0.76	1.65 ± 0.53	4.68 ± 0.21**	22.65 ± 0.36**	2.81 ± 0.74**
	Liver	1.38 ± 0.54	8.48 ± 0.53	0.99 ± 0.33	8.24 ± 0.42**	50.10 ± 1.95**	3.60 ± 0.46**
	Brain	0.65 ± 0.24	0.75 ± 0.11	1.05 ± 0.42	4.02 ± 0.46**	20.57 ± 1.36**	1.80 ± 0.42*
	Lung	1.77 ± 0.46	14.30 ± 0.43	0.70 ± 0.41	5.79 ± 0.29**	15.09 ± 3.24**	2.48 ± 0.69**
	Kidney	2.85 ± 0.45	4.05 ± 0.26	1.63 ± 0.18	3.98 ± 0.12**	27.54 ± 0.17**	2.84 ± 0.53**
	Plasma (*μ*g · mL^−1^)	1.67 ± 0.05	1.97 ± 0.16	1.78 ± 0.11	1.62 ± 0.07	2.87 ± 0.06**	2.68 ± 0.13**

1.0	Heart	4.26 ± 0.27	2.94 ± 0.24	2.11 ± 0.28	5.55 ± 0.31**	27.03 ± 0.31**	3.28 ± 0.43**
	Liver	1.66 ± 0.28	8.85 ± 0.51	1.30 ± 0.25	9.29 ± 0.72**	59.05 ± 3.74**	4.77 ± 0.42**
	Brain	0.81 ± 0.26	0.91 ± 0.89	1.21 ± 0.28	5.58 ± 0.68**	25.66 ± 2.69**	2.76 ± 0.63**
	Lung	1.82 ± 0.20	15.32 ± 0.64	0.92 ± 0.13	9.96 ± 0.66**	17.80 ± 1.25**	2.99 ± 0.17**
	Kidney	2.74 ± 0.33	3.67 ± 0.38	1.53 ± 0.22	3.38 ± 0.34**	16.93 ± 0.81**	2.43 ± 0.29**
	Plasma (*μ*g · mL^−1^)	1.29 ± 0.20	1.99 ± 0.06	1.90 ± 0.15	1.46 ± 0.05	2.18 ± 0.09**	2.42 ± 0.08**

3.0	Heart	3.31 ± 0.32	1.53 ± 0.45	1.14 ± 0.13	4.59 ± 0.52**	19.07 ± 1.16**	2.24 ± 0.54**
	Liver	0.89 ± 0.12	6.18 ± 0.59	0.69 ± 0.11	6.87 ± 0.61**	37.78 ± 3.43**	2.63 ± 0.81**
	Brain	0.48 ± 0.14	0.57 ± 0.20	0.63 ± 0.12	3.69 ± 0.84**	17.86 ± 2.60**	1.65 ± 0.23**
	Lung	1.51 ± 0.16	11.87 ± 0.71	0.31 ± 0.44	4.56 ± 0.75**	12.31 ± 1.46	1.53 ± 0.45**
	Kidney	1.94 ± 0.24	3.03 ± 0.23	1.15 ± 0.13	2.27 ± 0.28	10.16 ± 2.77**	1.61 ± 0.45
	Plasma (*μ*g · mL^−1^)	0.16 ± 0.06	0.98 ± 0.01	0.79 ± 0.02	0.49 ± 0.02	0.92 ± 0.01	0.87 ± 0.04

**P < 0.01 compared with *Panax notoginseng*.

**Table 6:** Apparent permeability coefficients (*P*_app_) of NGR1, GRg1, and GRe with or without the addition of 200 *μ*M Borneol on the Caco-2 Model.

Compound	*P*_app_ (apical to basolateral) (× 10^−7^ cm/s)	*P*_app_ (basolateral to apical) (× 10^−7^ cm/s)	*E*_*r*_
NGR1	0.64 ± 0.08	0.68 ± 0.12	1.06
GRg1	3.48 ± 0.42	3.64 ± 0.29	1.05
GRe	5.46 ± 0.40	5.73 ± 0.37	1.05
NGR1 + Borneol	1.87 ± 0.23**	1.95 ± 0.34**	1.04
GRg1 + Borneol	9.05 ± 0.67**	9.51 ± 0.62**	1.05
GRe + Borneol	12.67 ± 1.01**	13.65 ± 1.59**	1.08

*P < 0.05, **P < 0.01 compared with corresponding single compound such as NGR1, GRg1, or GRe.
